# Association between trough serum vancomycin concentration and vancomycin-associated acute kidney injury and 30-day mortality in critically ill elderly adults

**DOI:** 10.1186/s12879-024-09227-x

**Published:** 2024-03-20

**Authors:** Jialong Chen, Jing Lin, Jianzhen Weng, Yang Ju, Yanming Li

**Affiliations:** 1 Department of Pulmonary and Critical Care Medicine, Beijing Hospital, National Center of Gerontology, the Institute of Geriatric Medicine, Chinese Academy of Medical Sciences, Beijing, People’s Republic of China; 2 Graduate School, Peking Union Medical College, Chinese Academy of Medical Sciences, Beijing, China; 3 Department of Infectious Disease, Peking Union Medical College Hospital, Chinese Academy of Medical Sciences, Peking Union Medical College, Beijing, China

**Keywords:** Vancomycin trough concentration, Critically ill, Acute kidney injury, Mortality, Elderly

## Abstract

**Background:**

Vancomycin-associated acute kidney injury (VA-AKI) is the most clinically relevant side effect of vancomycin. The objective of this study was to investigate the association between VTC and VA-AKI as well as 30-day mortality in critically ill elderly adults.

**Method:**

Elderly patients with trough serum vancomycin concentration records(VTC) in the Medical Information Mart-IV (MIMIC-IV) and eICU databases were retrospectively studied.

**Results:**

A total of 3,146 critically ill elderly adults were finally enrolled. The incidence of VA-AKI in the elderly population was 76.5%. Logistic regression analysis revealed significant relationships between VA-AKI and various factors, including VTC, comorbidities, and laboratory indicators, and SOFA, and GCS score. For each mg/L increase, the OR for VA-AKI increased by 2.5%. The association between VTC and 30-day mortality was found to be statistically significant (odds ratio (OR): 1.021, 95% CI: 1.010–1.031), *P* < 0.001). The Restricted cubic splines (RCS) curves revealed that VTC ranged of 19.67 to 35.72 mg/l for AKI and 19.17 to 42.86 mg/l for 30-day mortality exhibit OR with 95% CI above 1, indicating statistically significant associations with an increased risk of AKI and 30-day mortality, respectively. In the subgroup analysis, VTC was identified as a risk factor for VA-AKI in specific patient groups, including white individuals, female patients, those with shock, patients with SOFA > 6, patients with baseline creatinine > 1.2 mg/dl and patients with or without exposed to other nephrotoxic medications.

**Conclusion:**

This study found the significant association between VTC and the incidence of VA-AKI and 30-day mortality in critically ill elderly adults. The RCS curves indicated concentration ranges for AKI (19.67–35.72 mg/L) and 30-day mortality (19.17–42.86 mg/L), signifying increased risk.

**Supplementary Information:**

The online version contains supplementary material available at 10.1186/s12879-024-09227-x.

## Introduction

Vancomycin, a glycopeptide antibiotic with a substantial bactericidal effect on gram-positive bacterial infections, is commonly used for empiric coverage of methicillin-resistant *Staphylococcus aureus* (MRSA), *Streptococcus sp*., *Enterococcus sp*., *Clostridium sp*., and *Eubacterium sp* [1]. It has also been recommended as the first-line treatment for MRSA infections by American, European, and Chinese guidelines [2–5]. In the USA, almost 10% of hospitalized patients (3.3 million patients) receive vancomycin treatment each year [6, 7].

Vancomycin-associated acute kidney injury (VA-AKI) is the most clinically relevant side effect of vancomycin. Previous studies found that 5%–43% of non-elderly patients exposed to vancomycin developed VA-AKI [8–10], which will significantly prolong hospital stays, increase mortality, morbidity, and cost, and may lead to chronic kidney disease [11].

Since the life expectancy of the general population has continued to increase, population aging is a nonnegligible global problem. Renal aging is one of the most noteworthy issues caused by aging. Renal aging can lead to renal function changes, such as a decline in glomerular filtration rate and sodium reabsorption, and renal structural changes, such as a decline in total nephron size and a decrease in nephron number [12]. Therefore, age-related changes in kidney function and structure and multiple comorbidities can increase the susceptibility of elderly adults to VA-AKI [10]. The pathophysiology of VA-AKI remains uncertain. VA-AKI has a dose‒response relationship, and the incidence of VA-AKI increases with higher vancomycin concentrations and doses [13]. Vancomycin is mainly excreted through the kidney. Researchers have found that elderly patients are at risk of VA-AKI [10]. Moreover, elderly people are often complicated with immune system dysfunction and are prone to gram-positive bacterial infections [14, 15]. However, there has been limited published literature on the association of VTC with VA-AKI and 30-day mortality in critically ill elderly patients.

Due to vancomycin nephrotoxicity, vancomycin therapy should be guided by therapeutic drug monitoring (TDM) to reduce VA-AKI risk and optimize effectiveness, particularly in elderly patients. Recent clinical guidelines recommend that vancomycin trough levels should be kept above 10 mg/l and target trough concentration levels should remain at 15–20 mg/l for serious infections caused by MRSA [2–5]. The objective of this study was to investigate the association between VTC and VA-AKI as well as 30-day mortality in critically ill elderly adults.

Method.

### Database

This study was performed using data from the Medical Information Mart for Intensive Care IV (MIMIC-IV) and the eICU collaborative research database [16, 17]. MIMIC-IV, a large, open, and public database, contains numerous anonymized patients who were admitted to the critical care units of Beth Israel Deaconess Medical Center (BIDMC) from 2008 to 2019. The eICU Database is a multicenter database comprising deidentified health data associated with over 200,000 admissions to ICUs across the United States between 2014 and 2015.

MIMIC and eICU databases include vital sign measurements, care plan documentation, comorbid diseases, laboratory tests, microbe findings, severity of illness measures, diagnosis information, and treatment information. The authors have completed ethics courses and gained access to the databases described above.

### Study population

The inclusion criteria included the following: 1) elderly patients (age ≥ 65 years), 2). with records of vancomycin treatment, 3). at least one vancomycin trough concentration(VTC) record. The exclusion criteria were as follows: 1) length of stay < 3 days, 2) duration of vancomycin < 2 days, 3) receiving dialysis or extracorporeal membrane oxygenation (ECMO) before vancomycin was administered, and 4) patients with related missing information included VTC record, serum creatinine, vancomycin record. Some patients with missing important information, including certain demographic details (e.g., age, gender), past medical history, blood creatinine levels and urine output for assessing kidney function, were excluded.

### Data extraction

The Structured Query Language (SQL) with the PostgreSQL tool (version 9.6) was used to extract data from the MIMIC-IV and eICU databases. The extracted information included age, sex, comorbidities, and laboratory tests. Comorbidities included diabetes, myocardial infarction, chronic pulmonary disease, renal disease, and cerebrovascular disease. We determined the VTC based on the maximum trough concentration observed after obtaining bacterial cultures and after vancomycin administration. During this period, laboratory tests were averaged for each of the patient's indicators. Based on the information above, we calculated sequential organ failure assessment (SOFA) and Glasgow Coma Scale (GCS) scores.

### Definition

The primary and secondary outcomes of this study were VA-AKI and 30-day mortality after vancomycin was administered, respectively. We used the 2012 Kidney Disease: Improving Global Outcomes (KDIGO) definition of AKI [18]: An increase in serum creatinine (SCr) by ≥ 0.3 mg/dl within 48 h or an increase in SCr to at least 1.5 times baseline within 1 week or continuous urine output < 0.5 ml/(kg*h). AKI patients were at least stage I in severity. Stage II was defined as a 2–2.9-fold increase in SCr from baseline or continuous urine output < 0.5 ml/(kg*h) for at least 12 h. Stage III was defined as a threefold increase in SCr from baseline, an increase in SCr to ≥ 4 mg/dl, receiving CRRT therapy, continuous urine output < 0.3 ml/(kg*h) for at least 24 h or anuria for at least 12 h. Other nephrotoxic medications included aminoglycosides, first-generation and second-generation cephalosporins, penicillin, tetracycline, rifampicin, Nonsteroidal Anti-Inflammatory Drugs(NSAIDs), amphotericin B, fluconazole, contrast agents, chemotherapeutic drugs like cisplatin and carboplatin, as well as immunosuppressive drugs such as cyclosporine and tacrolimus.

### Statistical analysis

Descriptive statistics were presented as either numbers and percentages for categorical variables or means and standard deviations for continuous variables. Continuous variables were compared using Student's t-test for pairwise comparisons and one-way ANOVA for comparisons across multiple groups, while categorical variables were compared pairwise with chi-square test. Regarding vancomycin therapeutic guidelines [5], we divided patients into 4 groups according to trough levels: ≤ 10.0 µg/ml, 10.0–15.0 µg/ml, 15.0–20.0 µg/ml, and > 20 µg/ml. Univariate and multivariate analyses for assessing independent risk predictors were performed using the logistic regression model. In the stepwise logistic regression model using the backwards selection approach, predictor variables were included if their *p*-values were less than 0.05 and removed if their *p*-values were greater than 0.10, based on the significance level for variable selection. Restricted cubic splines (RCSs) were employed to investigate the dose–response relationship between the VTC and outcomes, including VA-AKI and 30-day mortality. The RCS analysis was performed using the 25th, 50th, and 75th percentiles of the distribution of troughs as fixed knots. The variables utilized in the RCS analysis were selected based on their statistical significance in the prior multiple logistic regression analysis. Log-rank tests and Kaplan‒Meier curves were also performed. A two-sided *P* < 0.05 was considered statistically significant. R (version 4.2.1) software and SPSS software (v23.0; IBM, Armonk, NY) were used for statistical analyses.

## Results

### Characteristics of patients with different trough concentrations and outcomes

**Fig. 1:**
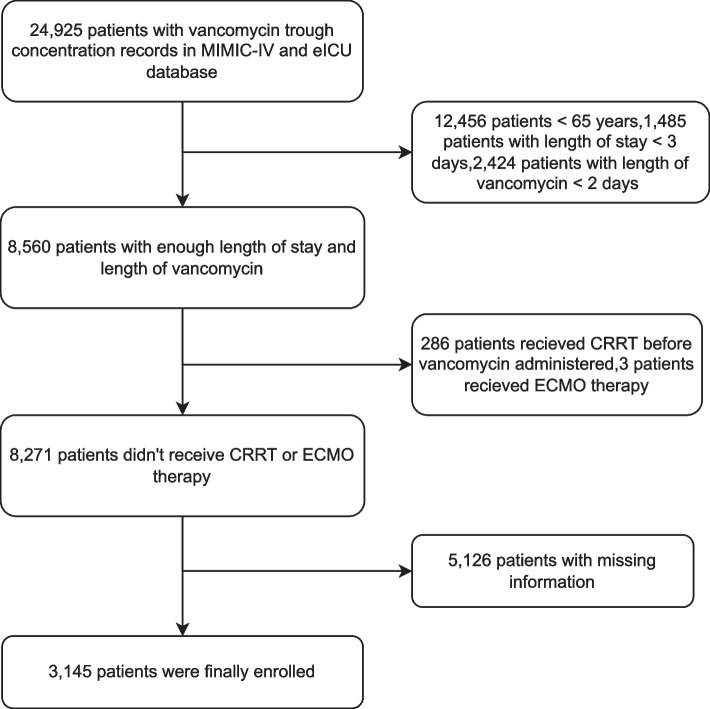
Flow diagram for patients recruitment in this study. MIMIC-IV = Multiparameter Intelligent Monitoring in Intensive Care IV

As shown in Fig. 1, a total of 3,145 critically ill elderly patients were enrolled for our final data analysis after screening by the inclusion and exclusion criteria. Table 1 showed the characteristics of patients among different VTC groups. White people accounted for most patients enrolled in this study. The mean ages were 77, 76, 75, and 75 for troughs of 5–10, 10.1–15, 15.1–20, and > 20(mg/l), respectively. Compared to other groups, more patients with troughs > 20 mg/l had comorbidities, including congestive heart failure, renal disease, and liver disease. Hemoglobin, serum bilirubin, and creatinine in the > 20 µg/ml group were higher than those in the other groups (*p* < 0.001). The proportions of patients exposed to other nephrotoxic medications were 13.31% in the 5–10 mg/l group, 13.49% in the 10–15 mg/l group, 22.02% in the 15–20 mg/l group, and 30.00% in the > 20 mg/l group(*p* < 0.001).The length of stay was also longer in the high vancomycin concentration group. The incidences of VA-AKI and 30-day mortality increased with vancomycin trough concentration levels. The VA-AKI rates were 64.0%, 72.5%, 73.7%, and 82.6% for troughs of 5–10 (mg/l), 10.1–15, 15.1–20, and > 20, respectively. The 30-day mortality rates were 14.4%, 15.9%, 17.4%, and 25.0% for troughs of 5–10, 10.1–15, 15.1–20, and > 20 (mg/l), respectively. The main distribution of pathogen species in patients is described in Supplemental Table S1.

**Table 1:** Characteristics of the patients according to vancomycin trough concentration

	**vancomycin trough concentration group**				*P* value
	≤ 10 ug/ml (*N* = 353)	10–15 ug/ml (*N* = 611)	15–20 ug/ml (*N* = 731)	> 20 ug/ml (*N* = 1450)	
Ethnicity,n(%)					0.247
White	264(74.8%)	464(75.9%)	572(78.2%)	1107(76.3%)	
Black(African American	27(7.6%)	42(6.9%)	58(7.9%)	133(9.2%)	
Other	62(17.6%)	105(17.2%)	101(13.8%)	210(14.5%)	
Male,n(%)	185(52.4%)	350(57.3%)	417(57.0%)	852(58.8%)	0.193
Age(year)	77 ± 8	76 ± 8	76 ± 7	75 ± 7	< 0.001
Comorbidity,n(%)					
Myocardial infarction,n(%)	21(5.9%)	60(9.8%)	65(8.9%)	171(11.8%)	0.006
Congestive heart failure,n(%)	48(13.6%)	111(18.2%)	131(17.9%)	393(27.1%)	< 0.001
Chronic pulmonary disease,n(%)	108(30.6%)	229(37.5%)	250(34.2%)	527(36.3%)	0.125
Cerebrovascular disease,n(%)	57(16.1%)	108(17.7%)	119(16.3%)	209(14.4%)	0.277
Hypertension,n(%)	144(40.79%)	234(38.30%)	274(37.48%)	511(35.24%)	0.104
Diabetes,n(%)	113(32.0%)	204(33.4%)	260(35.6%)	547(37.7%)	0.107
Renal disease,n(%)	57(16.1%)	108(17.7%)	156(21.3%)	361(24.9%)	< 0.001
Liver disease,n(%)	12(3.4%)	27(4.4%)	47(6.4%)	107(7.4%)	0.008
Malignant cancer,n(%)	61(17.3%)	107(17.5%)	135(18.5%)	268(18.5%)	0.918
Laboratory test					
White blood cell(*10^9^(l)	12.61 ± 6.31	12.89 ± 7.08	13.32 ± 9.63	13.22 ± 7.36	0.435
Hemoglobin( g/dl)	10.04 ± 1.58	9.82 ± 1.53	9.63 ± 1.48	9.43 ± 1.39	< 0.001
Platelet(*10^9^/l)	213.46 ± 104.82	216.57 ± 107.19	214.07 ± 103.16	221.63 ± 108.71	0.332
Bilirubin(mg/dl)	0.88 ± 1.01	0.98 ± 1.51	1.29 ± 2.23	1.41 ± 2.68	< 0.001
Albumin(g/dl)	2.61 ± 0.57	2.61 ± 0.60	2.62 ± 0.61	2.59 ± 0.56	0.796
Creatinine(mg/dl)	1.20 ± 0.91	1.28 ± 1.01	1.31 ± 0.90	1.46 ± 0.95	< 0.001
Glucose(mg/dl)	144.56 ± 41.36	148.21 ± 39.67	146.23 ± 38.06	144.95 ± 38.03	0.320
vancomycin trough concentration(mg/L)	7.30 ± 2.09	12.72 ± 1.47	17.52 ± 1.43	27.11 ± 7.41	< 0.001
GCS	5 ± 6	5 ± 6	5 ± 6	4 ± 5	< 0.001
SOFA	8 ± 4	9 ± 4	9 ± 4	10 ± 4	< 0.001
Length of stay(day)	12.43 ± 6.70	13.99 ± 7.88	14.81 ± 9.09	15.97 ± 9.63	< 0.001
Other nephrotoxic medications,n(%)	47(13.31%)	46(13.49%)	161(22.02%)	435(30%)	< 0.001
Ventilation,n(%)	191(54.1%)	294(48.1%)	348(47.6%)	529(36.5%)	< 0.001
Shock,n(%)	115(32.6%)	252(41.2%)	286(39.1%)	731(50.4%)	< 0.001
Outcome					
VA-AKI,n(%)	226(64.0%)	443(72.5%)	539(73.7%)	1198(82.6%)	< 0.001
30-day mortality,n(%)	51(14.4%)	97(15.9%)	129(17.4%)	362(25.0%)	< 0.001

Compared to the non-AKI group, more patients with VA-AKI had comorbidities, including myocardial infarction, congestive heart failure, renal disease, liver disease, and malignant cancer. As shown in Table 2, the vancomycin trough concentration of the VA-AKI group was higher than that of the non-AKI group (20.50 ± 8.65 vs. 17.81 ± 9.51, *p* < 0.001). The length of stay of the VA-AKI group was significantly longer than that of the non-AKI group (*p* < 0.001). Patients with VA-AKI were more likely to have higher SOFA scores and lower GCS scores, which suggested that the illness of patients in the AKI group was more severe than that of patients in the non-AKI group (*p* < 0.001). Among the non-AKI group, 45 patients (6.09%) were exposed to other nephrotoxic medications, whereas in the AKI group, 712 patients (29.59%) were exposed to such medications(*p* < 0.001). The 30-day mortality in the AKI group was higher than that in the non-AKI group (23.4% vs. 10.1%, *p* < 0.001).

**Table 2:** Characteristics of the VA-AKI patients and non-AKI patients

	Total (*N* = 3145)	Non-AKI (*N* = 739)	AKI (*N* = 2406)	*P* value
Ethnicity,n(%)				0.018
White	2407(76.5%)	594(80.4%)	1813(75.4%)	
Black/African American	260(8.3%)	50(6.8%)	210(8.7%)	
Other	478(15.2%)	95(12.9%)	383(15.9%)	
Male,n(%)	1804(57.4%)	405(54.8%)	1399(58.1%)	0.108
Age(year)	76 ± 7	76 ± 7	76 ± 7	0.764
Comorbidity,n(%)				
Myocardial infarction,n(%)	317(10.1%)	27(3.7%)	290(12.1%)	< 0.001
Congestive heart failure,n(%)	683(21.7%)	44(6.0%)	639(26.6%)	< 0.001
Chronic pulmonary disease,n(%)	1114(35.4%)	258(34.9%)	856(35.6%)	0.741
Cerebrovascular disease,n(%)	493(15.7%)	113(15.3%)	380(15.8%)	0.742
Hypertension,n(%)	1163(36.98%)	335(45.33%)	828(34.41%)	< 0.001
Diabetes,n(%)	1124(35.7%)	243(32.9%)	881(36.6%)	0.064
Renal disease,n(%)	682(21.7%)	98(13.3%)	584(24.3%)	< 0.001
Liver disease,n(%)	193(6.1%)	15(2.0%)	178(7.4%)	< 0.001
Malignant cancer,n(%)	571(18.2%)	156(21.1%)	415(17.2%)	0.017
Laboratory test				
White blood cell(*10^9/^l)	13.11 ± 7.79	12.96 ± 6.66	13.16 ± 8.11	0.548
Hemoglobin( g/dl)	9.62 ± 1.48	9.78 ± 1.58	9.58 ± 1.44	0.001
Platelet(*10^9^/l)	217.97 ± 106.72	209.26 ± 108.44	220.64 ± 106.06	0.011
Bilirubin(mg/dl)	1.24 ± 2.27	0.94 ± 1.18	1.33 ± 2.48	< 0.001
Albumin(g/dl)	2.60 ± 0.58	2.41 ± 0.54	2.66 ± 0.58	< 0.001
Creatinine(mg/dl)	1.36 ± 0.95	1.08 ± 0.61	1.45 ± 1.02	< 0.001
Glucose(mg/dl)	145.84 ± 38.75	147.66 ± 42.76	145.27 ± 37.42	0.143
Vancomycin trough concentration(mg/L)	19.86 ± 8.93	17.81 ± 9.51	20.50 ± 8.65	< 0.001
GCS	4 ± 5	7 ± 6	4 ± 5	< 0.001
SOFA	9 ± 4	7 ± 4	10 ± 4	< 0.001
Length of stay	14.92 ± 8.96	13.01 ± 8.48	15.51 ± 9.02	< 0.001
Other nephrotoxic medications,n(%)	757(24.07%)	45(6.09%)	712(29.59%)	< 0.001
Ventilation,n(%)	1362(43.3%)	485(65.6%)	877(36.5%)	< 0.001
Shock,n(%)	1384(44.0%)	217(29.4%)	1167(48.5%)	< 0.001
Outcome				
30-day mortality,n(%)	639(20.3%)	75(10.1%)	564(23.4%)	< 0.001

**Fig. 2:**
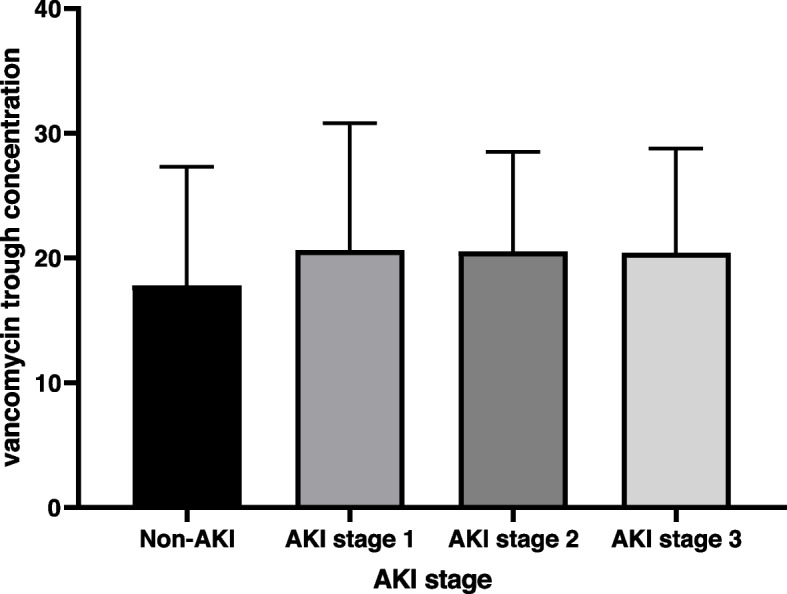
Association between vancomycin trough concentration and AKI stages

To further explore the association between troughs and VA-AKI, we compared concentrations in patients with different stages of acute kidney injury. As shown in Fig. 2, we did not find significant differences in stage 1, stage 2, and stage 3 (20.66 ± 10.17, 20.51 ± 8.00, 20.43 ± 8.36, *p* > 0.05).

### Univariate and multivariate analyses

Logistic regression analysis showed the possible risk factors and protective factors associated with VA-AKI (Table 3). Both univariate and multivariable logistic models showed that VTC, comorbidities including congestive heart failure, and laboratory indicators including platelet, baseline creatinine, and albumin, SOFA, other nephrotoxic medications, and GCS score were all related to the development of VA-AKI. For each 1 mg/l increase in VTC, the odds ratio (OR) for VA-AKI increased by 2.5% (OR = 1.025, 95% confidence interval (CI) = 1.010–1.040, *p* = 0.001). The risk factors associated with 30-day mortality include age, congestive heart failure, cerebrovascular disease, malignant cancer, white blood cell count, bilirubin levels, albumin levels, glucose levels, VTC, GCS score, and SOFA score(Table 4). Specifically, the VTC was found to have an OR of 1.018 with a 95% CI of 1.006 to 1.031, indicating a statistically significant association with 30-day mortality (*P* < 0.001).

**Table 3:** Analysis of the associations between AKI and vancomycin trough concentration

Univariable regression analysis				Multivariable regression analysis		
	*P* value	OR	95% CI	*P* value	OR	95% CI
vancomycin trough concentration	< 0.001	1.040	1.029–1.052	0.001	1.025	1.010–1.040
Age	0.764	1.002	0.991–1.013	-	-	-
Myocardial infarction	< 0.001	3.614	2.414–5.140	0.892	1.043	0.566–1.924
Congestive heart failure	< 0.001	5.712	4.157–7.849	0.003	2.254	1.323–3.840
Hypertension	< 0.001	1.580	1.337–1.868	0.143	1.191	0.943–1.506
Diabetes	0.064	1.179	0.990–1.404	0.061	1.264	0.990–1.614
Renal disease	< 0.001	2.097	1.662–2.644	0.710	1.069	0.754–1.515
Malignant cancer	0.017	1.284	1.045–1.577	0.972	1.202	0.940–1.536
Liver disease	< 0.001	0.779	0.634–0.957	0.203	1.526	0.796–2.924
White blood cell	0.549	1.003	0.992–1.015	-	-	-
Hemoglobin	0.001	0.914	0.866–0.966	0.916	0.996	0.923–1.085
Platelet	0.011	1.001	1.000–1.002	< 0.001	1.003	1.002–1.005
Bilirubin	0.001	1.138	1.056–1.226	0.672	1.018	0.937–1.058
Albumin	< 0.001	2.166	1.814–2.585	< 0.001	1.560	1.256–1.887
Creatinine	< 0.001	1.851	1.620–2.115	< 0.001	1.597	1.308–1.849
Other nephrotoxic medications	< 0.001	6.482	4.736–8.873	< 0.001	3.017	1.964–4.635
GCS	< 0.001	0.890	0.877–0.903	0.011	0.970	0.947–0.993
SOFA	< 0.001	1.155	1.130–1.180	< 0.001	1.424	1.080–1.209
Shock	< 0.001	2.266	1.898–2.705	0.007	1.025	1.010–1.040

**Table 4:** Analysis of the associations between 30-day mortality and vancomycin trough concentration

	Univariable regression analysis			Multivariable regression analysis		
	OR	95% CI	*P* value	OR	95% CI	*P* value
Ethnicity	1.047	(0.933 ~ 1.174)	0.439			
Gender	0.955	(0.803 ~ 1.136)	0.603			
Age	1.026	(1.015 ~ 1.038)	< 0.001	1.035	(1.021 ~ 1.048)	< 0.001
Myocardial infarction	1.498	(1.152 ~ 1.950)	0.003	0.956	(0.710 ~ 1.288)	0.769
Congestive heart failure	2.001	(1.652 ~ 2.425)	< 0.001	1.662	(1.310 ~ 2.109)	< 0.001
Chronic pulmonary disease	1.108	(0.927 ~ 1.324)	0.260			
Cerebrovascular disease	1.354	(1.082 ~ 1.694)	0.008	1.579	(1.236 ~ 2.018)	< 0.001
Hypertension	1.221	(1.017–1.467)	0.033	0.808	(0.634–1.028)	0.083
Diabetes	0.960	(0.802 ~ 1.150)	0.660			
Renal disease	1.554	(1.277 ~ 1.892)	< 0.001	1.012	(0.795 ~ 1.289)	0.920
Malignant cancer	1.486	(1.205 ~ 1.833)	< 0.001	1.644	(1.300 ~ 2.079)	< 0.001
Liver disease	2.237	(1.644 ~ 3.044)	< 0.001	1.299	(0.904 ~ 1.866)	0.157
AIDS	0.540	(0.066 ~ 4.399)	0.565			
White blood cell	1.026	(1.015 ~ 1.037)	< 0.001	1.019	(1.008 ~ 1.031)	0.001
Hemoglobin	0.855	(0.803 ~ 0.910)	< 0.001	0.962	(0.897 ~ 1.032)	0.276
Platelet	0.998	(0.997 ~ 0.999)	< 0.001	0.999	(0.998 ~ 1.000)	0.083
Bilirubin	1.152	(1.104 ~ 1.202)	< 0.001	1.074	(1.026 ~ 1.123)	0.002
Albumin	0.701	(0.590 ~ 0.832)	< 0.001	0.586	(0.481 ~ 0.714)	< 0.001
Creatinine	1.329	(1.224 ~ 1.443)	< 0.001	1.099	(0.990 ~ 1.219)	0.076
Glucose	1.004	(1.002 ~ 1.007)	< 0.001	1.004	(1.002 ~ 1.007)	< 0.001
Vancomycin trough concentration	1.033	(1.023 ~ 1.043)	< 0.001	1.018	(1.006 ~ 1.031)	< 0.001
GCS	0.917	(0.898 ~ 0.936)	< 0.001	0.936	(0.913 ~ 0.959)	< 0.001
Ventilation	0.925	(0.778 ~ 1.099)	0.375			
Other nephrotoxic medications	1.461	(1.204–1.773)	< 0.001	0.111	(0.608 ~ 1.053)	0.800
Shock	2.437	(2.043 ~ 2.908)	< 0.001	1.147	(0.893 ~ 1.474)	0.282
SOFA	1.170	(1.145 ~ 1.196)	< 0.001	1.099	(1.062 ~ 1.137)	< 0.001

### Restricted cubic splines

**Fig. 3:**
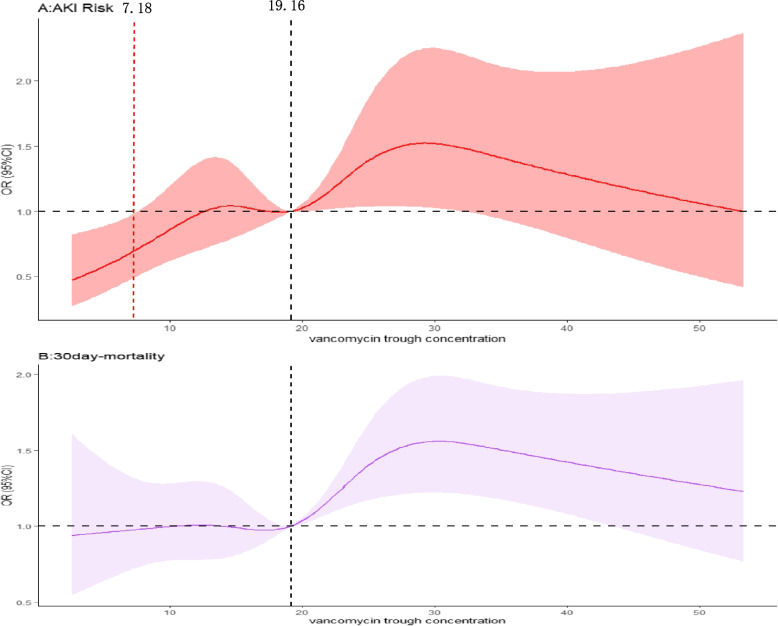
Dose–response relationships between vancomycin trough concentration and the outcomes. **A**, **B** represent the occurrence of AKI,and 30-day mortality

The identified risk factors of VA-AKI and 30-day mortality above were taken into account as covariates during the RCS analysis to investigate the linear dose–response relationship between the VTC and the outcomes, as illustrated in Fig. 3. The RCS model showed a linear dose‒response association between the vancomycin trough concentration and outcomes, including VA-AKI and 30-day mortality (Fig. 3). In Fig. 3A, the *P* value for nonlinearity was 0.08, and the *P* value for nonlinearity was 0.06 in Fig. 3B. In this study, the RCS curves revealed that VTC ranged of 19.67 to 35.72 mg/l for AKI and 19.17 to 42.86 mg/l for 30-day mortality exhibit OR with 95% CI above 1.

### Kaplan‒Meier analysis

**Fig. 4:**
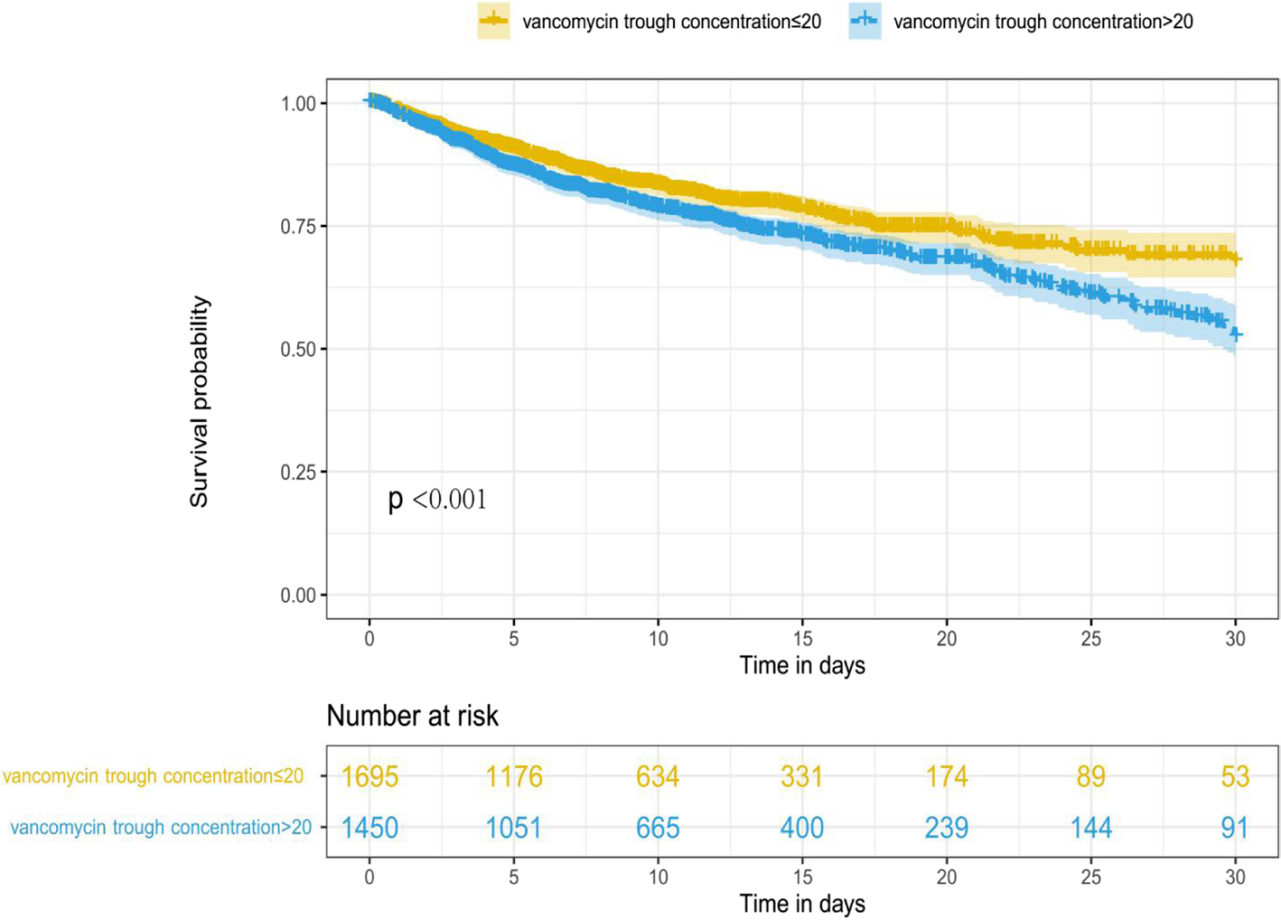
Survival analysis between different vancomycin trough concentration levels

A significant difference was observed between patients with trough concentrations ≤ 20 and those with trough concentrations > 20 for all-cause 30-day mortality (10.1% vs. 23.4%, Fig. 4).

### Subgroup analyses

**Fig. 5:**
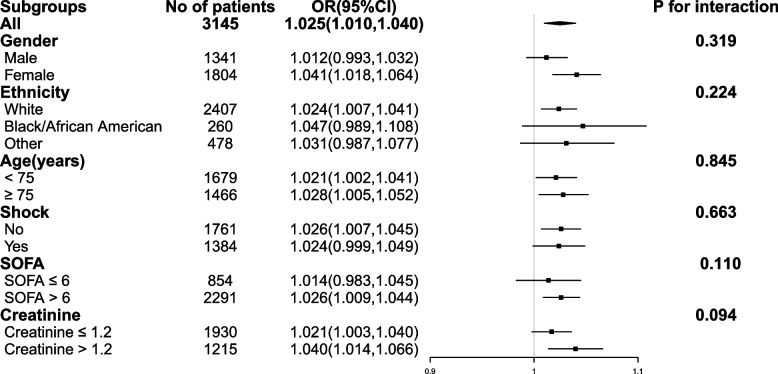
Subgroup analysis of the associations between the occurs of VA-AKI and vancomycin trough concentration (continuous variable)

Subgroup analyses were performed for sex (male or female), age (< 75 or ≥ 75 years), race (white, black/African American or other), SOFA score (< 6 or ≥ 6), shock (with or without), baseline creatinine (creatinine ≤ 1.2 µg/ml or creatinine > 1.2 µg/ml) and exposed to other nephrotoxic medications (yes or not). As shown in Fig. 5, VTC was still a risk factor in white people(OR 1.024,95%CI1.007,1.041), female patients(OR 1.041,95% CI 1.018–1.064), patients without shock(OR 1.026,95% CI 1.007–1.045), patients with SOFA > 6(OR 1.026,95%CI1.009–1.044) and patients with baseline creatinine > 1.2 mg/dl(OR 1.040,95% CI 1.014–1.066). In both the age < 75 and age ≥ 75 groups, the risks of VA-AKI increased with trough concentration (OR 1.021, 95% CI 1.002–1.041, OR 1.028, 95% CI 1.005–1.052, respectively). In with the exposed to other nephrotoxic medications and without exposed to other nephrotoxic medications, the risks of VA-AKI increased with trough concentration (OR 1.09, 95% CI 1.02–1.16, OR 1.02, 95% CI 1.01–1.04, respectively).

## Discussion

In this study, we found that the incidence of VA-AKI in the elderly population was 76.5%, which was significantly higher than that in non-elderly patients. We found a 64.0% rate of VA-AKI if the trough was < 10 mg/l compared to rates of 72.5% for troughs of 10–15 mg/l, 73.7% for 15–20 mg/l, and 82.6% for > 20 mg/l (*P* < 0.05). Furthermore, for each mg/l increase, the OR for VA-AKI increased by 2.5%. A linear relationship between trough levels and VA-AKI levels was also observed in this study.

Logistic regression analysis revealed VTC was significantly associated with VA-AKI and 30-day mortality.

The exact pathophysiological mechanisms of VA-AKI are not yet fully understood. Oxidative stress is considered the primary mechanism of VA-AKI. When intracellular drug concentrations in the renal tubules of patients with risk factors are high, oxidative stress can lead to an imbalance between reactive oxygen species and antioxidants, altered mitochondrial function, and cellular apoptosis in proximal renal tubules. Vancomycin-associated renal tubular cast and allergic reaction are other possible mechanisms involved in VA-AKI [10]. Significant eosinophil infiltration was found in VA-AKI patients' kidney biopsy results [19]. It is proposed to be associated with the T-cell-mediated type-4 delayed hypersensitivity reaction or probable complement system activation [20, 21].

Due to narrow therapeutic index of vancomycin, careful dosing and monitoring are critical to ensure therapeutic efficacy while minimizing the risk of adverse events such as nephrotoxicity. Monitoring VTC allows for dosage adjustments to maintain concentrations within a range that is effective yet below nephrotoxic levels. This is particularly important in vulnerable populations, such as the elderly and those with pre-existing renal impairment. The recognition of VTC as a critical factor in vancomycin therapy aligns with existing clinical guidelines. Emerging evidence suggests that monitoring the area under the concentration–time curve (AUC) to minimum inhibitory concentration (MIC) ratio may be superior to trough concentration monitoring alone for predicting vancomycin efficacy and toxicity [22]. However, AUC monitoring is not yet universally available or feasible in all settings, making VTC monitoring a vital and more accessible tool for guiding vancomycin therapy.

Even if the pathophysiological mechanisms are unclear, the incidence of VA-AKI remains high. Previous studies have shown that almost 5%–43% of patients exposed to vancomycin will develop VA-AKI, which VA-AKI is more common than renal failure caused by other antibiotics [11, 23, 24]. Elderly people are at high risk of VA-AKI. A retrospective study enrolled elderly patients and was conducted in China [25]. Among 647 patients, 102 patients had confirmed VA-AKI, with an incidence of 15.8%, which differed widely from ours. Potential reasons are as follows: first, patients enrolled in our study were older. The mean age of the patients in this study was 76 years, and the mean age in the previous study was 71 years; Second, the elderly adults included are mainly white people. In our subgroup analysis, we did not find that trough concentration was an independent risk factor for VA-AKI in nonwhite people; Third, patients in our study were from intensive care units and emergency departments. We had a higher proportion of patients complicated with shock (44% vs. 11.6%). This means that the patients included in our study were in worse health status and had more serious illnesses. The severity of infection and ICU residence impact the development of AKI in patients exposed to vancomycin [26].

Numerous studies have recognized the relationship between trough levels and VA-AKI [8, 27–29]. A previous meta-analysis indicated that a cutoff of 15 mg/L detected VA-AKI with a sensitivity of 62.6% and a specificity of 65.5%, while applying a 20 mg/L threshold resulted in a sensitivity of 42.9% and a specificity of 82.5% [30]. Horey et al. found nephrotoxicity rates of 5%, 3%, 11%, 24%, and 82% for maximal troughs of 5–10 mg/L, 10.1–15, 15.1–20, 20.1–35, and > 35, respectively [31]. Similarly, Kassem et al. found that the incidences of AKI in vancomycin trough < 10, 10 ≤ vancomycin trough < 15, 15 ≤ vancomycin trough < 20, and vancomycin trough ≥ 20 mg/L subgroups were 8.02, 13.61, 13.70 and 31.82%, respectively [32]. Guillaume et al. found that a serum vancomycin level greater than 40 mg/L (OR 3.75; 95% CI, 1.40–10.37) was associated with VA-AKI. In another study, vancomycin nephrotoxicity occurred in 42 patients (29.6%) with trough concentrations > 15 mg/ml and 13 (8.9%) with trough concentrations of < 15 mg/ml. Multivariate regression analysis also showed that vancomycin trough concentrations of > 15 mg/ml were a risk factor for nephrotoxicity [33]. Previous studies also highlighted the need for careful vancomycin dosing to balance treatment efficacy and nephrotoxicity risk in enterococcal infections [34]. An adult trough concentration ≥ 15 mg/L improves outcomes but increases nephrotoxicity, while an AUC/MIC ratio ≥ 400 mg·h/L aligns with better responses [35]. Pediatric dosing requires lower trough levels compared to adults, emphasizing age-specific monitoring and adjustments to optimize therapeutic effectiveness and minimize renal harm [36].

There is no consensus on the exact threshold of renal injury caused by vancomycin trough concentrations, as this threshold depends on a variety of factors, such as the patient's age. For elderly patients (aged ≥ 80 years), a previous study also indicated that vancomycin trough concentrations ≥ 15 mg/L were independent risk factors leading to nephrotoxicity [37]. Moreover, vancomycin trough concentrations (initial or maximal trough concentration) may be different in different articles about VA-AKI. Some researchers only considered the initial trough level. However, vancomycin therapy should be guided by TDM, and the initial trough concentration may differ widely from subsequent trough levels. Since vancomycin is mainly excreted through the kidney, reduced kidney function from any cause will lead to an elevated trough level. The risk of VA-AKI increases with trough concentrations. In our opinion, the maximal trough level was more appropriate. In this study, we found that the risks of AKI significantly increased when the trough concentration was > 19.67 mg/L. In contrast, if the trough concentration was < 7.8, the risk of VA-AKI did not increase with the trough concentration. We observed significant differences in trough concentration between non-AKI patients and AKI patients (17.81 ± 9.51 vs. 20.50 ± 8.65, *p* < 0.001) but no differences in trough concentration among different AKI-stage patients.

In addition to vancomycin concentration, congestive heart failure was an independent risk factor for VA-AKI (OR 2.254, 95% CI 1.323–3.840). However, we did not find an association between renal diseases and VA-AKI (OR 0.905, 95% CI 0.638–1.284). Instead, baseline creatinine was closely related to subsequent VA-AKI. It is suggested that patients with kidney disease that leads to renal dysfunction are susceptible to VA-AKI. To reduce the effect of other confounding factors such as exposure to other nephrotoxic drugs, age, and disease severity, we performed a multifactorial regression analysis and found risk factors for AKI including VTC. The severity of illness and infection are well-known risk factors for VA-AKI. In our study, SOFA and shock were proven to be risk factors for VA-AKI (OR 1.208,95% CI 1.142–1.279; OR 1.025, 95% CI 1.010–1.040, respectively). The identified risk factors of VA-AKI and 30-day mortality above were taken into account as covariates during the RCS analysis and an linear dose–response relationship between the VTC and the outcomes was observed.

Similar to Pan’s study [25], the serum albumin level was also observed as an independent protective factor. The protective effects of albumin are alleviating oxygen stress and oxidative damage and binding or delivering protective lysophosphatidic acid [38, 39]. Another laboratory test, platelet count, was also associated with VA-AKI. The potential mechanism may be impaired renal microvascular circulation due to a decreased blood flow rate by microthrombi [40, 41].

The relationship between trough concentration and 30-day mortality was also explored. We found that the trough level was associated with 30-day mortality. When the trough concentration is > 19.17 mg/L, the risk of 30-day mortality will significantly increase. Increasing concentrations of vancomycin cannot enhance the ability of bacterial killing but significantly increase the risks of VA-AKI [42]. In this study, we found that increasing concentrations may also increase the risk of 30-day mortality.

In conclusion, this study found the significant association between VTC and the incidence of VA-AKI and 30-day mortality in critically ill elderly adults. The RCS curves indicated concentration ranges for AKI (19.67–35.72 mg/L) and 30-day mortality (19.17–42.86 mg/L), signifying increased risk.

There were some limitations in this study. First:retrospective study design. The study employed a retrospective design, which may introduce information bias and missing data. Data extracted from medical information databases could be subject to issues of data quality and accuracy. Second: data source. The study utilized data from the Medical Information Mart-IV (MIMIC-IV) and eICU databases, which may represent specific patient populations. Generalizability of the findings might be limited to other settings or patient cohorts. Third: potential confounding factors. Even though we used multiple regression analyses, retrospective studies may not account for all potential confounding factors that could influence the occurrence of VA-AKI and the study outcomes. Forth: sample size. Although the study includes a substantial number of critically ill elderly patients, the sample size may still be limited for some subgroup analyses. Fifth, we did not investigate the relationship between the vancomycin regimen and VA-AKI. Sixth: microbiological outcomes: Our study did not extensively explore microbiological outcomes, such as the rate of eradication of specific pathogens and the development of resistance. Seventh:Prospective Validation: The findings from our study should be validated in prospective studies to confirm the identified risk factors and associations with clinical outcomes. Finally:long-term outcomes: Our study focused on short-term outcomes, specifically VA-AKI and 30-day mortality. Further studies could investigate the long-term consequences of vancomycin-associated AKI in the elderly.

### Supplementary Information

**Supplementary Material 1.**

## Data Availability

No datasets were generated or analysed during the current study.
