# Supplementary material for: Association between trough serum vancomycin concentration and vancomycin-associated acute kidney injury and 30-day mortality in critically ill elderly adults
Source: BMC Infect Dis. 2024 Mar 20;24:330. doi: 10.1186/s12879-024-09227-x (PMC10953182; doi:10.1186/s12879-024-09227-x)
Supplement: Supplementary file 1 — Supplementary Material 1. [file 12879_2024_9227_MOESM1_ESM.docx]

| Supplemental Material Table 1 Distribution of pathogens species in patients | | |
| --- | --- | --- |
|  | Pathogens | Number |
| 1 | *STAPH* *AUREUS* *COAG* *+* | 642( 19.5%) |
| 2 | *STAPHYLOCOCCUS,* *COAGULASE* *NEGATIVE* | 419( 12.7%) |
| 3 | *METHICILLIN* *RESISTANT* *STAPH* *AUREUS* | 338( 10.3%) |
| 4 | *ENTEROCOCCUS* *SP.* | 297(9.0%) |
| 5 | *CLOSTRIDIUM* *DIFFICILE* | 147(4.5%) |
| 6 | *MIXED* *BACTERIAL* *FLORA* | 112(3.4%) |
| 7 | *STAPHYLOCOCCUS* *EPIDERMIDIS* | 67(2.0%) |
| 8 | *CORYNEBACTERIUM* *SPECIES* *(DIPHTHEROIDS)* | 63( 1.9%) |
| 9 | *ENTEROCOCCUS* *FAECIUM* | 63( 1.9%) |
| 10 | *OTHER* | 1142(34.7%) |
